# Designing Efficient Metal-Free Dye-Sensitized Solar Cells: A Detailed Computational Study

**DOI:** 10.3390/molecules28176177

**Published:** 2023-08-22

**Authors:** Fatma M. Mustafa, Ahmed A. Abdel Khalek, Abdulla Azzam Mahboob, Mahmoud K. Abdel-Latif

**Affiliations:** 1 Chemistry Department, Faculty of Science, Beni-Suef University, Beni-Suef City 62521, Egypt; dr_fatma238@yahoo.com (F.M.M.); ahmedakhalek41@gmail.com (A.A.A.K.); 2 Chemistry Department, Collage of Science, United Arab Emirates University, Al-Ain P.O. Box 15551, United Arab Emirates; abdulla.mahboob@uaeu.ac.ae

**Keywords:** dye-sensitized solar cells, D-π-A configuration dyes, adsorption on titanium(IV) hydroxide, dye-iodine interaction, TDDFT calculations

## Abstract

The modulation of molecular characteristics in metal-free organic dyes holds significant importance in dye-sensitized solar cells (DSSCs). The D-π-A molecular design, based on the furan moiety (π) in the conjugated spacer between the arylamine (D) and the 2-cyanoacrylic acid (A), was developed and theoretically evaluated for its potential application in DSSCs. Utilizing linear response time-dependent density functional theory (TDDFT) with the CAM-B3LYP functional, different donor and acceptor groups were characterized in terms of the electronic absorption properties of these dyes. All the studied dye sensitizers demonstrate the ability to inject electrons into the semiconductor’s conduction band (TiO_2_) and undergo regeneration through the redox potential triiodide/iodide (I_3_^−^/I^−^) electrode. TDDFT results indicate that the dyes with CSSH anchoring groups exhibit improved optoelectronic properties compared to other dyes. Further, the photophysical properties of all dyes absorbed on a Ti(OH)_4_ model were explored and reported. The observed results indicate that bidentate chemisorption occurs between dyes and TiO_4_H_5_. Furthermore, the HOMO–LUMO energy gaps for almost all dye complexes are significantly smaller than those of the free dyes. This decrease of the HOMO–LUMO energy gaps in the dye complexes facilitates electron excitation, and thus more photons can be adsorbed, guaranteeing larger values of efficiency and short-circuit current density.

## 1. Introduction

In recent times, the demand for efficient and environmentally friendly energy sources has become increasingly important. This has led to a growing interest in the development of renewable energy systems. Around twenty years ago, considerable efforts were directed towards developing energy sources that convert sunlight into electricity through charge separation in molecular systems. One particularly effective technology in this domain is dye-synthesized solar cells (DSSCs) [1], which offer cost-effectiveness and environmental sustainability. These devices utilize various chemical materials, taking into account both energy considerations and structural aspects [2]. In DSSCs, a dye sensitizer is adsorbed onto a semiconductor surface, typically TiO_2_ with a wide band gap [3,4,5,6,7,8,9,10,11].

Among the different phases of TiO_2_, anatase is known to be the most stable and exhibits excellent photocatalytic activity [12,13]. When the dye is optically excited, rapid electrons generated by light are injected into the conduction band (CB) of TiO_2_, and the oxidized dye is subsequently reduced by the redox electrolyte present in the cell [14]. Traditionally, efforts have focused on achieving the highest certified light conversion efficiency (PCE) for various dyes. For instance, the ruthenium-based dye N719 achieved a PCE of 11.4% [15], the porphyrin-based dye reached 11.5% [16], and an organic sensitizer combined with an iodide-based electrolyte achieved 10.2% [17]. More recently, a higher PCE value of 14.5% was accomplished by utilizing a cobalt-based redox electrolyte and combining organic co-sensitizers with an alkoxysilyl anchor dye, ADEKA-1, and a carboxyl anchor dye, LEG [9].

For a considerable period, the triiodide/iodide (I_3_^−^/I^−^) redox shuttle has been widely regarded as the preferred mediator for efficiently reducing oxidized dyes in DSSCs [15,16,17,18]. The iodide/triiodide redox couple offers a significant advantage by preventing the recombination of electrons injected into the titanium dioxide with the oxidized dye and triiodide, leading to a prolonged charge-separated state lifetime [19]. The reaction between the iodide/triiodide redox pair has been extensively studied [20,21] with isolated dyes for the purpose of dye regeneration. The regeneration process of the oxidized dye employs various elemental mechanisms. Extensive research has been conducted to enhance the PCE of DSSCs, including the development of molecular sensitizers for improved light harvesting efficiency [22,23,24,25] and the discovery of suitable redox electrolytes to enhance the open-circuit voltage [26,27].

The proper selection of a favorable dye binding arrangement is crucial for comprehending the behavior of electron transfer. Various adsorption configurations for the carboxyl anchor group on the TiO_2_ surface have been reported, including monodentate, bidentate chelating, and bidentate bridging modes [28]. Among these, the bidentate bridging adsorption configuration has been employed in recent studies. This decision was made due to the system’s high stability and the nature of charge separation. [1,28,29]. Density functional theory calculations have been extensively employed to investigate dye adsorption on the TiO_2_ surface [2]. Organic photosensitizers, or metal-free dyes, have garnered significant attention from researchers in this field. These dyes have demonstrated the ability to capture a substantial portion of the solar spectrum, ranging from ultraviolet to near-infrared regions, resulting in impressive PCE values of around 14.55%.

Seven N,N’-diphenyl-aniline (NNdPA) dyes based on the D−π–A architecture have been designed and analyzed by Leszczynski et al. [30] using cluster and periodic density functional theory (DFT) methodologies for utilization in dye-sensitized solar cells (DSSCs). Triphenylamine (TPA), an excellent electron donor widely used in organic photovoltaic functional materials, prevents aggregation due to its non-planar structure [31]. It also showed very good hole-transport properties. Triphenylamine-based dyes are extensively applied in DSSCs and showed high conversion efficiencies. Yanagida and co-workers first introduced the triphenylamine unit as an electron donor in organic dyes and obtained overall DSC efficiencies of 3.3% and 5.3% for dyes 1 and 2, respectively [32,33].

**Scheme 1:**
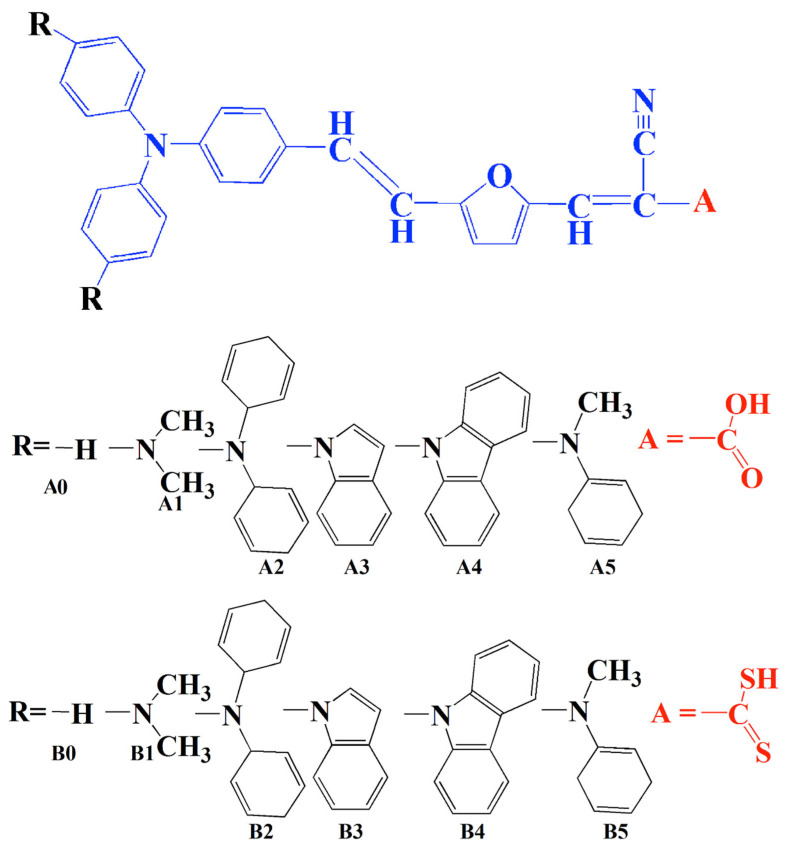
Molecular structures of the studied dyes, in which A0 (reference dye) is the anchoring group; A1–A5 dyes are COOH; and B0–B5 dyes are CSSH.

In this study, we examined the influence of modifying five donor groups and two anchoring groups on the geometries and optoelectronic properties of twelve organic dyes with a D-π-A structure, as illustrated in Scheme 1, using the methods DFT and TD-DFT. The reference dye (A0) has furan, 2-ethenyl (2-vinyl furane moiety), acting as a conjugated spacer between the arylamine donor and the 2-cyanoacrylic acid acceptor, as shown in Scheme 1. Ming-Chang et al. studied this dye and reported that it exhibits very high power conversion efficiencies (7.36%) [34]. The selection of these donor moieties is substantiated by their remarkable performance in similar DSSCs [1,2]. We systematically examined the consequences of substituting the studied donor units with two distinct anchoring groups, as depicted in Scheme 1, on the optoelectronic attributes. We meticulously devised a set of five A series dyes (A0–A5) with the carboxyl group (COOH) as the anchoring moiety and an additional set of six B series dyes (B1–B5) with the sulfhydryl group (CSSH) as the anchoring component (The designation A0 denotes the Reference dye). Furthermore, we present an analysis of the interactions between all the investigated dyes and a simplified model of the titanium oxide surface (TiO_4_H_5_), encompassing considerations of geometries and optoelectronic features.

## 2. Theoretical Background

The ionization potential (*IP*) and electron affinity (*EA*) serve as descriptors for the energy barriers of holes and electrons. A lower *IP* indicates an enhanced ability to create holes [35]. Conversely, a higher *EA* enhances the ability to accept electrons [36]. In solar cells, two primary parameters dictate the Power Conversion Efficiency (*PCE*): the short-circuit current density (*J_SC_*) and the open-circuit photovoltage (*V_OC_*). The *PCE* can be defined using Equation (1) [37,38]:

(1) $$PCE=FF\frac{\;\;J_{sc}\times V_{oc}}{P_{in}}\;\times \;100\%\;$$

where *FF* is the fill factor and *P_in_* is the incident solar power on the cell. The value of (*J_SC_*) in DSSCs is determined as follows [39]:

(2) $$J_{\mathrm{SC}}=q\;\int LHE\left(\lambda \right)\times \phi_{injection\;}\times \eta_{collection}\;d\lambda$$

In Equation (2), (q) represents the charge of an electron, (*LHE*) denotes the light-harvesting efficiency, (η*_collection_*) signifies the charge collection efficiency (which can be assumed constant for the same DSSC with different sensitizers), and (ϕ_*injection*_) represents the electron injection efficiency. To maximize the short-circuit current density (*J_SC_*), it is crucial for the *LHE* of the dyes to be as high as possible. The calculation of the *LHE* for the investigated dyes is determined using Equation (3), as described in references [40,41].

*LHE* = 1 − 10^−*f*^ (3)

In Equation (3), the variable *f* represents the oscillator strength associated with the wavelength λ_max_ of the dye. For DSSC applications, it is crucial for the dye sensitizer to possess high light-harvesting efficiency (*LHE*) values. The open-circuit photovoltage (*V_*OC*_*) is influenced by the electron injection from the excited dye to the conduction band of the semiconductor [42]. The electron injection ratio is affected by both the low *V*_*OC*_ and the short-circuit current density (*J_sc_*) in DSSCs. The relationship between the energy level of the lowest unoccupied molecular orbital (*E_LUMO_*) and *V_OC_* is described by the following equation:

(4) $$V_{\mathrm{OC}}=E_{\mathrm{LUMO}}-E_{CB}^{TiO_{2}}\;$$

where *E*_LUMO_ is the LUMO energy of the dye and $E_{CB}^{TiO_{2}}$ is the conduction band energy of the semiconductor (TiO_2_). It is difficult to accurately determine $E_{CB}^{TiO_{2}}$ because it is highly sensitive to the operating conditions, such as the pH of the solution. So, we have used $E_{CB}^{TiO_{2}}$ = −4.0 eV, in which this energy is an experimental value corresponding to conditions where the semiconductor is in contact with aqueous redox electrolytes of fixed pH 7.0. To obtain high (*J_sc_*) with high (***f***), (*LHE*), and (φ*_injection_*), this is related to the Δ*G_inject_* driving force of the electron injection from excited dye to TiO_2_ surface, which can be calculated by using the following equation [43]:

(5) $$\Delta G_{inject}=E_{dye}^{*}-E_{CB}^{TiO_{2}}\;$$

where $E_{dye}^{*}$ is the oxidized potential energy of the excited dye, as follows:

(6) $$E_{dye}^{*}=E_{dye}^{\;}-\;E\;$$

where, $E_{dye}^{\;}$ is the oxidation potential energy of the dye in the ground state, while *E* is the lowest vertical electronic excitation energy, corresponding to *λ_max_*. The dye regeneration (Δ*G_regen_*) can be calculated by the equation [44]:

(7) $$\Delta G_{regen}=E_{Redox}^{\;}-E_{dye}^{\;}\;$$

$E_{Redox}^{\;}$ is the ground-state oxidation potential of the triiodide/iodide redox couple electrolyte redox potential (−4.80 eV) [45].

### Computational Details

**Figure 1:**
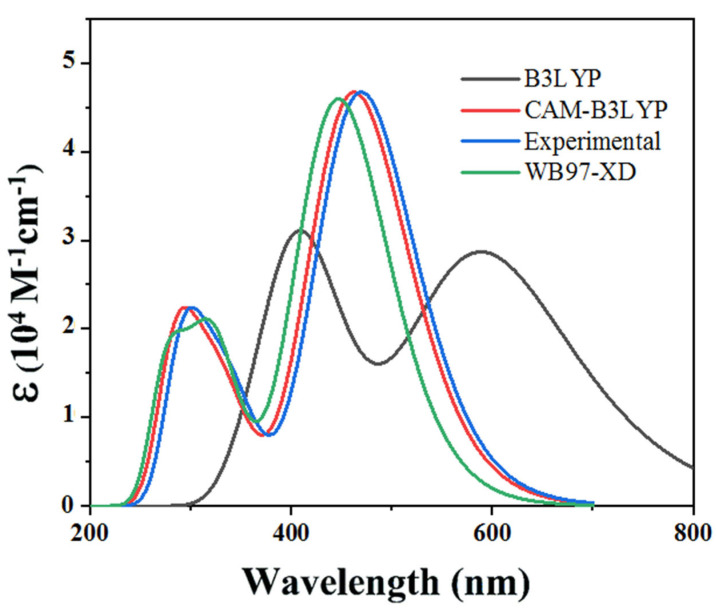
Calculated λ_max_ values of A0 at B3LYP, wB97XD, and CAM−B3LYP functions combined with the 6−31+G (d,p) basis set in comparison with the experimental value.

Density functional theory (DFT) calculations were performed to obtain the optimized structures of the molecules under investigation. The calculations were carried out using the G09 program [46] at the B3LYP/6-31+G (d,p) level of theory [47,48]. In fact, these molecules showed multiple conformations, and we chose the one previously reported for the diphenylamine donor [1]. Following optimization, frequency calculations were performed to confirm the minimum energy structure on the potential energy surface. TD-DFT calculations were employed to calculate the electronic absorption spectra for the twenty-five lowest singlet vertical excitations [49]. There is a wealth of literature showing that various functionals can yield accurate results in predicting the absorption spectra of organic molecules [50,51,52]. In this study, we deployed different functionals, namely B3LYP, wB97XD, and CAM-B3LYP, to be tested using the 6-31+G (d,p) basis set. Most of the calculations were performed in the gas phase, as preliminary results indicated that solvent simulations had only a slight effect on the results. However, to account for solvation effects, the optimized gas-phase geometries were re-optimized using the same functional and basis set for the calculation of the absorption spectra in tetrahydrofuran (THF). The obtained results are presented in Figure 1, where it is evident that the CAM-B3LYP functional value (λ_max_ = 462) exhibited good agreement with the experimental value (λ_max_ = 469) [34]. Therefore, the CAM-B3LYP functional in conjunction with the 6-31+G (d) basis set was used for TD-DFT calculations in this study. To account for solvent effects on the electronic absorption spectra, the self-consistent reaction field-polarizable continuum model (SCRF-PCM) [53] was employed. Additionally, other electronic properties such as light harvesting efficiency (LHE), ionization potentials (IPs), electronic affinities (EAs), open-circuit voltage (V_OC_), and global properties were computed to provide insights into the photophysical properties of these organic sensitizers.

## 3. Results and Discussion

### 3.1. Energetics and Distribution of Frontier Molecular Orbitals

**Figure 2:**
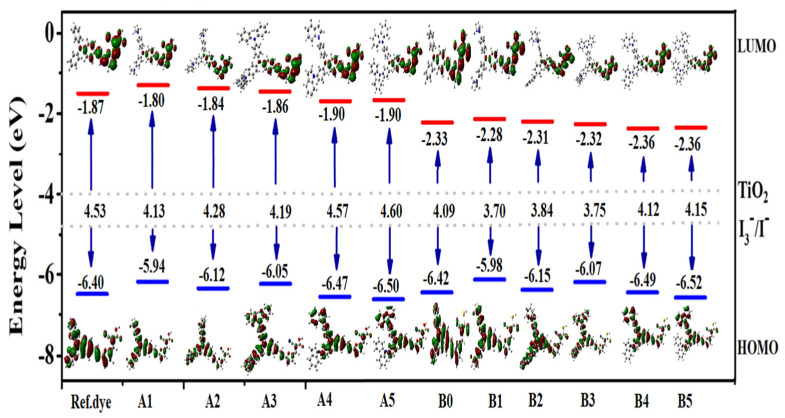
Frontier molecular orbitals and HOMO–LUMO energy levels for all studied organic sensitizers.

The distribution of the Frontier Molecular Orbitals (FMOs) determines the charge transfer characteristics. By comparing the relative positions of the highest occupied molecular orbital (HOMO) and lowest unoccupied molecular orbital (LUMO) energy levels, the ability to gain or lose an electron during redox processes can be assessed qualitatively [54]. In order for charge injection from the excited dye to occur, the LUMO of the dye should be positioned above the conduction band (CB) of the TiO_2_ semiconductor (−4.0 eV) to provide the necessary energy [55]. Additionally, for dye regeneration through electron acceptance from the electrolyte, the HOMO level of the dye should be below the redox potential of the iodide/triiodide electrolyte (−4.8 eV) [56]. Figure 2 illustrates the molecular energy levels of the twelve dyes, providing further insights into the influence of different donor and anchoring groups on the energy levels of HOMO, LUMO, and the energy gap (HOMO–LUMO). The reference dye (experimentally tested) and its designed derivatives demonstrate suitable HOMO and LUMO energy levels, making them suitable candidates as photosensitizers. Table 1 summarizes the HOMO, LUMO, and HOMO–LUMO energies of the dyes. Most of the designed derivatives exhibit lower LUMO energies compared to the reference dye, and dyes containing a CSSH anchoring group generally have lower LUMO levels than those with a COOH group. The LUMOs predominantly arise from either acceptor or anchoring groups. A previous investigation [57] highlighted the substantial role of anchoring groups in LUMOs, crucial for effective electron injection into the semiconductor interface. Our findings align well with those of Jyh-Chiang et al. [6], who reported significant LUMO contributions from their designed dyes featuring CSSH anchoring groups compared to COOH anchoring groups. The COOH anchoring groups’ contribution to LUMOs is merely 10%, whereas the CSSH anchoring group’s contribution is notably higher at 50%. Therefore, we expect that dyes with CSSH anchoring groups will have a strong electronic coupling with the semiconductor surface and can more easily inject excited electrons into the surface than dyes with COOH groups. Moreover, the LUMO energy levels of the designed dyes align better with the CB of TiO_2_, as they are more negative than those of the reference dye. This indicates a stronger driving force for electron injection, as measured by the difference between the LUMO and the conduction band edge of TiO_2_, which is expected to be more significant for the designed dyes compared to the reference dye. Furthermore, the majority of the designed dyes have higher HOMO energy levels than the reference dye, implying that their HOMO levels are closer to the redox potential of the electrolyte, facilitating the regeneration of the designed dyes. The HOMO levels of some designed dyes, such as A4, A5, B0, B4, and B5, are lower in energy compared to the reference dye. This can affect the energy gap of these designed dyes, causing it to be larger than that of the reference dye, as observed in A4 and A5. On the other hand, the energy gaps of the B0–B5 dyes exhibit a significant decrease compared to the A1–A5 dyes. The visualization of the HOMO and LUMO distributions over different parts of the molecule provides insights into the corresponding charge separation, as depicted in Figure 2. It can be observed that all designed molecules have a charge distribution similar to that of the reference dye, which is a successful photosensitizer that has been extensively tested experimentally.

It has been observed that as the acceptor strength intensifies, the LUMO level experiences a downward shift, whereas the HOMO level remains relatively stable due to its predominant localization on the donor and π-bridge parts. This delocalization of the LUMO orbital facilitates the attachment of the dye to the TiO_2_ semiconductor. Based on the previous studies [58,59] we expect that the sensitizers with the smallest HOMO–LUMO energy gap values will show a higher efficiency, and hence the designed dyes with CSSH anchoring groups may exhibit a better performance than the others. The calculated HOMO–LUMO gaps for the dyes with a CSSH anchoring group are lower than those with a COOH group, which is responsible for the redshift in the UV/vis absorption spectra of the dyes with CSSH anchoring groups.

### 3.2. Absorption Properties

**Figure 3:**
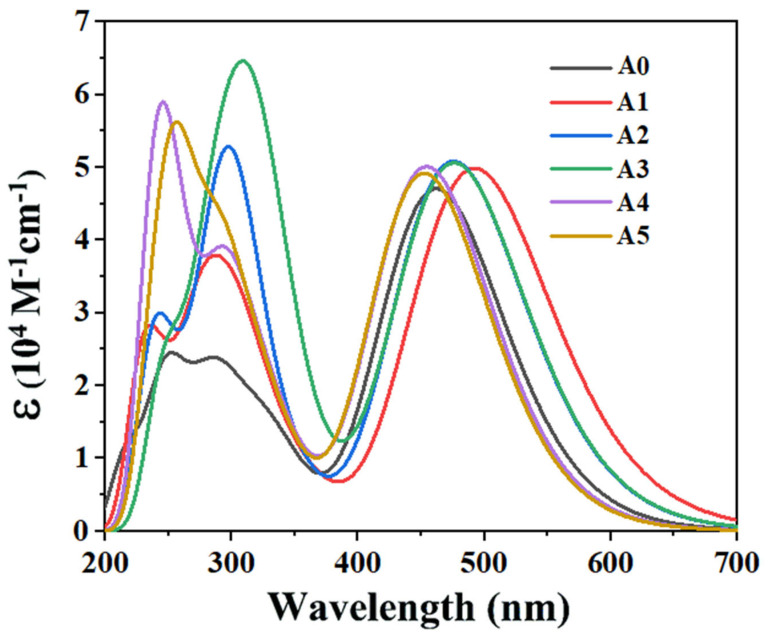
Absorption spectra of A1–A5 dyes in THF, calculated by TD−DFT at the CAM−B3LYP/6-31+G (d,p) level of theory.

**Figure 4:**
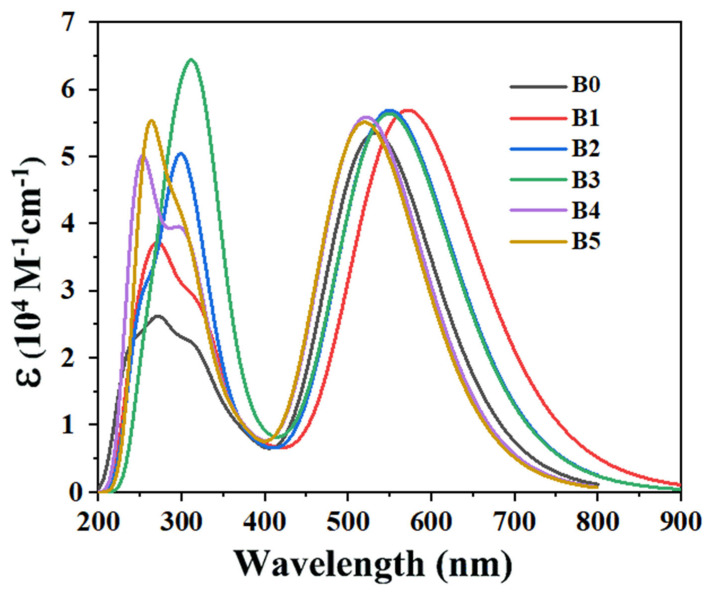
Absorption spectra of B0–B5 dyes in THF, calculated by TD−DFT at the CAM−B3LYP/6-31+G (d,p) level of theory.

Table 2 provides the absorption wavelengths of the two main bands and their intensities for the investigated dyes, calculated at the CAM−B3LYP/6−31G+(d) level. The absorption spectra of the reference dye (A5) and dyes B0-B5 are presented in Figure 3 and Figure 4, respectively. These calculations reveal two allowed excited states in the ultraviolet-visible (UV-Vis) region, characterized by large transition probabilities. The first peak is a sharp, long-wavelength absorption band with a higher intensity, occurring in the range of 450 to 490 nm for A1–A5 and 520 to 530 nm for B0–B5. This peak can be attributed to intramolecular charge transfer (IACT) from donor to acceptor units. The second, weaker absorption band appears in the range of 250 to 350 nm for all studied dyes and is attributed to π → π* electronic transitions. In A1 to A3 dyes, a red shift compared to the reference dye can be observed due to substitution in the donor region. On the other hand, in A4 and A5, a blue shift is observed relative to the reference dye. Changing the COOH group to CSSH as an anchoring group affects the absorption spectra of B0-B5 dyes; the longer-wavelength band exhibits variations in the sequence B1 > B2 > B3 > B0 > B4 > B5. The red shift of the absorption spectrum observed in A1, A2, A3, B0, B1, B2, B3, B4, and B5 compared to the reference dye indicates a better match with the solar photon flux spectrum. This alignment with the solar spectrum is beneficial for enhancing the short-circuit current density (J_sc_) and, consequently, the power conversion efficiency (PCE) of solar cells. Thus, the incorporation of CSSH as an anchoring group is expected to improve the light-harvesting properties, as reported below.

### 3.3. Electron Injection, Dye Regeneration, Open-Circuit Voltage, and Light Harvesting Efficiency

#### 3.3.1. Electron Injection and Dye Regeneration

The complete cycle occurs when electrons are transferred from the excited dyes’ highest occupied molecular orbital (HOMO) to their lowest unoccupied molecular orbital (LUMO) and injected into the conduction band of the TiO_2_ semiconductor. This cycle results in the regeneration of the dyes into their ground state. The potential gradient between the electrolyte and the oxidized dyes is the major driving force for the dye’s regeneration in the ground state. A sufficiently large potential gradient enhances the regeneration of the ground state. It also helps reduce the recombination rate between the oxidized dye and the conduction band of the TiO_2_ semiconductor. The values of ΔG_injection_, as presented in Table 3, demonstrate that the excited state of the dyes is positioned above the conduction band of TiO_2_, enabling rapid injection of electrons from the excited state to the semiconductor. The ΔG_injection_ values of the investigated dyes follow the order: A1 (−0.58 eV) < A3 (−0.55 eV) < A2 (−0.49 eV) < A4 (−0.26 eV) < A5 (−0.24 eV) < B3 (−0.19 eV) = B1 (−0.19 eV) < B2 (−0.11 eV) < B0 (0.09 eV) < B4 (0.11 eV) < B5 (0.13 eV). Therefore, the driving force for electron injection from the oxidized dye to the conduction band of TiO_2_ follows the order: B5 > B4 > B0 > B2 > B1 = B3 > A5 > A4 > A2 > A3 > A1. This indicates that A1 has the smallest ΔG_inj_ value, while B5 has the largest value, with a significant driving force for electron injection into TiO_2_ [58]. Our results reveal that the driving force for electron injection is remarkably large for the investigated dyes, facilitating electron transfer from the dye to the conduction band of TiO_2_. Additionally, for a fast electron transfer, the free energy of regeneration (ΔG_reg_) of the dye should be minimized. Therefore, the driving forces for regeneration (ΔGreg) of the investigated dyes follow the order: B5 (1.72 eV) < A5 (1.7 eV) < B4 (1.69 eV) < A4 (1.67 eV) < B0 (1.62 eV) < B2 (1.35 eV) < A2 (1.32 eV) < B3 (1.27 eV) < A3 (1.25 eV) < B1 (1.18 eV) < A1 (1.14 eV). Higher voltages and power conversion efficiencies are expected for the designed dyes compared with the Reference dye.

#### 3.3.2. Open Circuit Voltage

The highest performance in terms of Jsc (short-circuit current density) and V_OC_ (open-circuit voltage) values leads to the best performance of a specific sensitizer in a Dye-Sensitized Solar Cell (DSSC). V_OC_ provides information about the mobility and number of charge carriers across the interface. A high V_OC_ value indicates minimal loss due to charge recombination, thus improving cell efficiency. The calculated V_OC_ values for the designed dyes range from 1.64 eV to 2.20 eV. Increasing V_OC_ leads to higher J_SC_ values. Among the studied dyes, A1, A2, and A3 exhibit the highest V_OC_, resulting in higher JSC values compared to other dyes. Additionally, A1, A2, and A3 may exhibit slower charge recombination compared to other dyes. This can be attributed to their higher HOMO (highest occupied molecular orbital) energy levels. Consequently, the energy difference between the HOMO of these dyes and the redox potential of the I^3−^/I^−^ couple is lower, leading to a slower dye regeneration process. This slower regeneration rate causes a back reaction at the TiO_2_-dye-electrolyte interface [58]. This trend of the V_oc_ values reveals the effects of incorporating the different donor groups (A1, A2, and A3) as far as preventing π-aggregation and charge recombination.

#### 3.3.3. Light Harvesting Efficiency (LHE)

Light harvesting efficiency (LHE) is another important factor related to the efficiency of Dye-Sensitized Solar Cells (DSSCs). Table 2 shows the calculated LHEs. In this case, LHE refers to the ability of the dye sensitizer to efficiently harvest light through intramolecular charge transfer transitions. A larger LHE value indicates a higher light-harvesting capability, which in turn maximizes the photocurrent response [60]. The investigated dyes exhibit LHE values ranging from 0.941 to 0.960, compared to 0.931 for the reference dye. This indicates that the designed dyes can provide a photocurrent similar to or even larger than that provided by the reference dye. Additionally, we observed that the calculated oscillator strength for all the studied dyes is greater than that of the reference dye. The increased oscillator strength is expected to enhance the LHE value and improve the overlap with the solar spectrum, particularly in the visible range.

### 3.4. Adsorbed Dyes on the Model of Titanium Hydroxide

#### 3.4.1. Optimized Geometry

The interaction between a dye and TiO_2_ nanoparticles leads to the hybridization of molecular orbitals, resulting in changes in the relative energy level alignment. To account for this effect, the combined dye-TiO_2_ system is considered in this study. The bidentate bridging mode is adopted to model the adsorption configuration of the dye on Ti(OH)_4_. This mode is chosen for its superior stability compared to the monodentate ester-like configuration, which has been experimentally and theoretically predicted [61,62]. According to the proposal by Hilal et al. [63] and others [60,64,65], the adsorption of the dye on the TiO_2_ surface can be modeled using a Ti(OH)_3_H_2_O moiety. In this moiety, the titanium atom is octahedral, with two oxygen atoms from the dye’s anchor group occupying positions. The acidic hydrogen from the dye relocates to the titanium complex [55]. The fully optimized geometry of the dye-Ti(OH)_4_ complexes is depicted in Figure S1 (Supporting Information). The adsorption onto the titanium oxide surface has a limited effect on the dye’s geometry, primarily affecting the anchor group and its neighboring bonds. The O-C-O bond angle decreases by only 3–5°, and the S-C-S bond angle decreases by only 3–6°. Additionally, the CO and CS bond lengths are shortened by no more than 0.07 Å. The calculated bond lengths between two carboxylate oxygen atoms and titanium atoms on the surface for dyes A1–A5 are very similar, around 2.13 and 2.15 Å for the five complexes. This is in agreement with previous theoretical studies and indicates bidentate chemisorption [66]. The calculated bond lengths between two sulfur atoms and titanium atoms on the surface for dyes B0–B5 are also very similar, approximately 2.35 and 2.42 Å for the six complexes, as shown in Figure S1. Due to proton transfer from the hydroxyl oxygen and sulfur atoms of the dye to the Ti surface, the Ti-O lengths of the dye-Ti(OH)_4_ complexes vary.

#### 3.4.2. Frontier Molecular Orbitals (FMOs) of All Studied Dyes Adsorbed onto Ti(OH)_3_H_2_O

**Figure 5:**
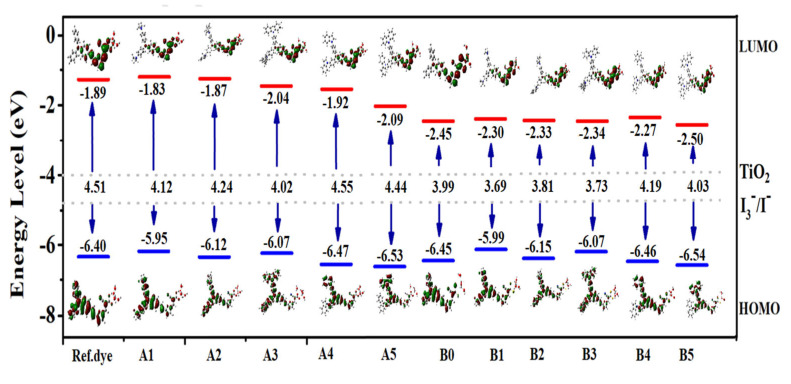
Frontier molecular orbitals and HOMO–LUMO energy levels for all studied organic sensitizers adsorbed onto Ti(OH)_3_H_2_O (Isodensity contour of 0.02 a.u.).

The Frontier Molecular Orbital (FMO) energies of all dyes attached to Ti(OH)_4_ have been calculated and summarized in Figure 5. It is observed that the HOMO and LUMO of the dye complexes are mostly unaffected by combined states and do not exhibit mixed molecular orbital (MO) character [67]. The HOMO, which is primarily localized on the dye, remains largely unaffected by the interaction with TiO_2_ and retains the HOMO character of the free dye. On the other hand, the LUMO of the complexes is distributed among the dye anchor groups, with a slight distribution on parts of the titanium oxide, as shown in Figure 5. The EHOMO, ELUMO, and energy gap (HOMO–LUMO) values of all the studied dyes attached to Ti(OH)_4_ are provided in Table 4. Upon complexation, the FMO energies of the investigated dyes exhibit significant variations. The HOMO energy levels of dyes A1, A3, A5, B0, B1, and B5 increase (become more negative), making them less suitable for the dye regeneration process with the electrolyte layer. However, the HOMO energy level of B4 decreases (becomes less negative), while the HOMO energy levels of A2, A4, B2, and B3 remain constant. On the other hand, the LUMO energy levels for all dyes increase (become more negative), making them more favorable for electron injection (photoinduced electron transfer from the dye in the excited state) to the semiconductor surface. This indicates stronger electronic coupling between the LUMO of the dye and the TiO_2_ surface. Furthermore, the HOMO–LUMO energy gaps for almost all dye complexes are significantly smaller than those of the free dyes. This decrease of the HOMO–LUMO energy gaps in the dye complexes facilitates electron excitation, and thus more photons can be adsorbed, guaranteeing larger values of *J*_sc_ and *η* [33].

#### 3.4.3. Absorption Properties

**Figure 6:**
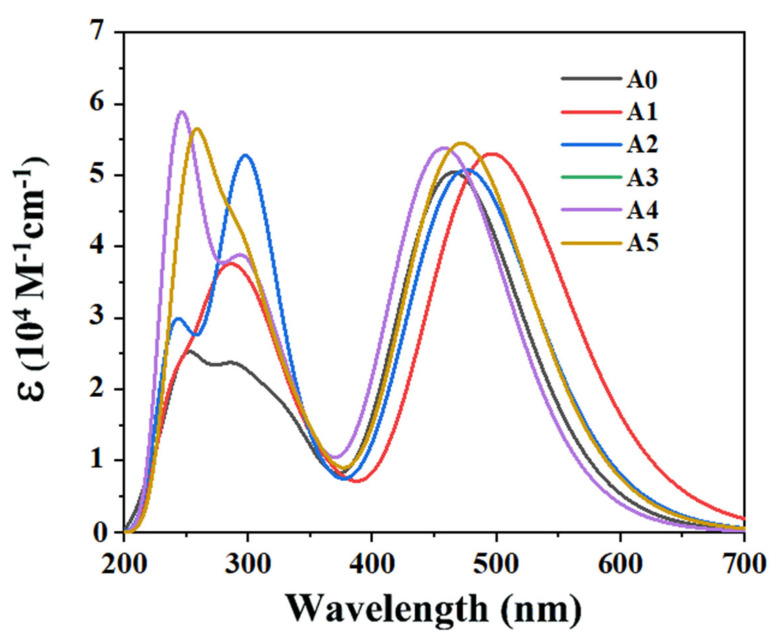
Absorption spectra of A0−A5 dyes adsorbed onto Ti(OH)_3_H_2_O in THF.

**Figure 7:**
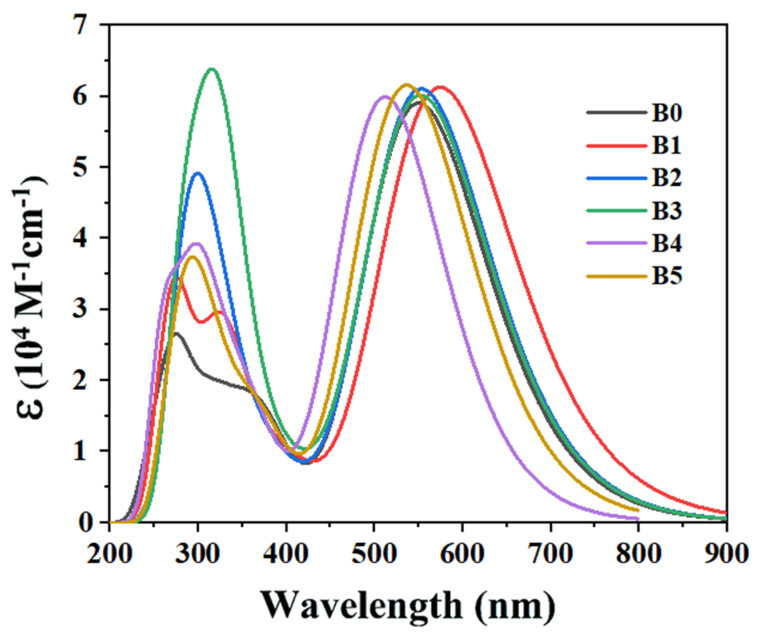
Absorption spectra of B0−B5 dyes attached and adsorbed onto Ti(OH)_3_H_2_O in THF.

The electronic absorption spectra of all the investigated dyes adsorbed onto Ti(OH)_3_H_2_O were calculated in THF solvent and are presented in Figure 6 and Figure 7. Table 5 provides the absorption wavelengths of the two intense bands and their intensities for the dye-Ti(OH)_4_ complexes. When compared to the free dyes, the dye-TiO_2_ complexes exhibit intensified absorption bands that are slightly red-shifted, except for B4, which shows an intensified and slightly blue-shifted absorption band. The intensification of the absorption bands in the dye complexes is advantageous for absorbing and utilizing more sunlight [59]. However, experimental studies have reported a blue shift in the absorption maximum for the dye complex compared to the free dye [62,63]. As mentioned in Table 3, the excitation energy decreases after attaching the designed dyes to TiO_2_. This can be attributed to the fact that the dye’s LUMO is higher than that of TiO_2_, while the HOMO level is above that of TiO_2_. These results indicate that the current dyes are efficient and effective in photo-current conversion, allowing them to absorb more energy from the solar spectrum. It is evident that the designed dye derivatives exhibit high photovoltaic efficiency and improved photoelectric performance as dye-sensitizers for DSSC applications.

## 4. Conclusions

The reference molecule used in this study is an experimentally well-characterized dye with a furan moiety as a conjugated spacer between the arylamine donor and the 2-cyanoacrylic acid acceptor, following a D-π-A configuration. The available experimental data for the reference dye were utilized to validate the employed DFT and TDDFT functions and to assess the potential of the twelve newly developed dyes, also following a D-π-A configuration but containing different donor and anchoring groups, as organic photosensitizers for DSSC applications. The DFT and TDDFT methods were employed to investigate the geometry, electronic, and optical properties of both the developed dyes and the reference dye. The results indicate that the developed dyes possess suitable energetic and spectroscopic parameters. Specifically, their LUMO energy levels are significantly higher than the conduction band of TiO_2_, while their HOMO energies are lower than the redox potential of the I^−^/I_3_^−^ system. The theoretical findings predict the geometries, electronic and photophysical parameters, absorption spectra, driving force, electron injection, electron regeneration, and oxidation potential energy of the designed dyes. These results demonstrate the effectiveness and excellence of the dye photosensitizers in DSSCs. Notably, the study reveals that the dye-TiO_2_ complex systems exhibit more stable and closely related geometry properties, suggesting a significant interaction between the dye and TiO_2_, which facilitates successful electronic injection and regeneration. In conclusion, the developed and derived organic dyes exhibit favorable characteristics and demonstrate improved performance in terms of photovoltaic energy and high photovoltaic energy sensitizer efficiency for solar cells. These dyes hold promise as important and novel dye sensitizers for solar cells.

## Figures and Tables

**Table 1:** Ground states E_HOMO_, E_LUMO_, and E_g_ of A0 (Reference dye), A1–A5, and B0–B5.

Dyes	E_HOMO_ (eV)	E_LUMO_ (eV)	Energy Gap (E_g_) (eV)	IP (eV)	EA (eV)
**A0**	−6.40	−1.87	4.53	6.40	1.87
**A1**	−5.94	−1.80	4.13	5.94	1.8
**A2**	−6.12	−1.84	4.28	6.12	1.84
**A3**	−6.05	−1.86	4.19	6.05	1.86
**A4**	−6.47	−1.90	4.57	6.47	1.90
**A5**	−6.50	−1.90	4.60	6.50	1.90
**B0**	−6.42	−2.33	4.09	6.42	2.33
**B1**	−5.98	−2.28	3.70	5.98	2.28
**B2**	−6.15	−2.31	3.84	6.15	2.31
**B3**	−6.07	−2.32	3.75	6.07	2.32
**B4**	−6.49	−2.36	4.12	6.49	2.36
**B5**	−6.52	−2.36	4.15	6.52	2.36

**Table 2:** Calculated excitation energy (E), absorption wavelength ($\lambda_{max\;}^{abs}$), oscillator strength (*f*), light harvesting efficiency (*LHE*), and the open-circuit voltage *(V*_oc_) of all studied dyes.

Dyes	Excitation Wavelength		Oscillator Strength (f)
	Energy (eV)	$\lambda_{max\;\;\;}^{abs}\;\;\;\;\;{\;}_{\;}(\mathrm{nm})$	
**A0**	2.68	461.91	1.16
**A1**	2.51	492.13	1.23
**A2**	2.60	475.59	1.25
**A3**	2.60	476.17	1.24
**A4**	2.72	454.29	1.23
**A5**	2.74	452.49	1.21
**B0**	2.33	531.93	1.32
**B1**	2.16	571.40	1.42
**B2**	2.25	549.74	1.40
**B3**	2.25	549.27	1.39
**B4**	2.37	521.73	1.38
**B5**	2.38	519.50	1.36

**Table 3:** Free energies of the charge injection ΔG_inj_ and dye regeneration ΔG_reg_ and the open-circuit voltage *(V*_oc_); all values are in eV.

Dyes	ΔG_inj_	ΔG_reg_	V_OC_	LHE
**A0**	−0.28	1.6	2.13	0.931
**A1**	−0.58	1.14	2.20	0.941
**A2**	−0.49	1.32	2.16	0.944
**A3**	−0.55	1.25	2.14	0.943
**A4**	−0.26	1.67	2.10	0.941
**A5**	−0.24	1.7	2.10	0.938
**B0**	0.09	1.62	1.67	0.952
**B1**	−0.19	1.18	1.72	0.970
**B2**	−0.11	1.35	1.69	0.960
**B3**	−0.19	1.27	1.68	0.959
**B4**	0.11	1.69	1.64	0.958
**B5**	0.13	1.72	1.64	0.957

**Table 4:** Ground states E_HOMO_, E_LUMO_, and E_g_ of all dye complexes.

Dyes	E_HOMO_ (eV)	E_LUMO_ (eV)	Energy Gap (E_g_) (eV)
**A0**	−6.40	−1.89	4.51
**A1**	−5.95	−1.83	4.12
**A2**	−6.12	−1.87	4.24
**A3**	−6.07	−2.04	4.02
**A4**	−6.47	−1.92	4.55
**A5**	−6.53	−2.09	4.44
**B0**	−6.45	−2.45	3.99
**B1**	−5.99	−2.30	3.69
**B2**	−6.15	−2.33	3.81
**B3**	−6.07	−2.34	3.73
**B4**	−6.46	−2.27	4.19
**B5**	−6.54	−2.50	4.03

**Table 5:** Calculated excitation energy (E), absorption wavelength ($\lambda_{max\;}^{abs}$*),* and oscillator strength (*f*) of all studied dyes adsorbed onto Ti(OH)_3_H_2_O.

Dyes	Excitation Wavelength		Oscillator Strength (f)
	Energy (eV)	$\lambda_{max\;\;\;}^{abs}\;\;\;\;\;{\;}_{\;}(\mathrm{nm})$	
**A0**	2.66	465.00	1.24
**A1**	2.49	496.04	1.31
**A2**	2.58	479.58	1.34
**A3**	2.49	479.07	1.34
**A4**	2.71	457.51	1.33
**A5**	2.62	471.97	1.34
**B0**	2.25	550.00	1.45
**B1**	2.15	574.59	1.51
**B2**	2.24	553.00	1.50
**B3**	2.24	552.29	1.48
**B4**	2.42	512.01	1.47
**B5**	2.31	536.65	1.52

## Data Availability

Data of the quantum chemical calculations are available upon request from the authors.

## Supplementary Materials

The following supporting information can be downloaded at: https://www.mdpi.com/article/10.3390/molecules28176177/s1, Figure S1: The optimized geometry of all designed dyes attached with the Ti(OH)4 surface.
