# Supplementary material for: Designing Efficient Metal-Free Dye-Sensitized Solar Cells: A Detailed Computational Study
Source: Molecules. 2023 Aug 22;28(17):6177. doi: 10.3390/molecules28176177 (PMC10488533; doi:10.3390/molecules28176177)
Supplement: Supplementary file 1 [file molecules-28-06177-s001.zip › molecules-2551225-supplementary.pdf]

molecules

MDPI

Supporting information

Article

Designing Efficient metal-free dyes Dye-Sensitized Solar Cells: a detailed computational study

F. M. Mustafa [1], Ahmed A. Abdel Khalek [1], Abdulla Azzam Mahboob [2], and Mahmoud K. Abdel-Latif [1,2]

[1]Chemistry Department, Faculty of Science, Beni-Suef University, Beni-Suef City, Egypt
[2]Chemistry Department, Collage of Science, United Arab Emirates University, Al-Ain, UAE

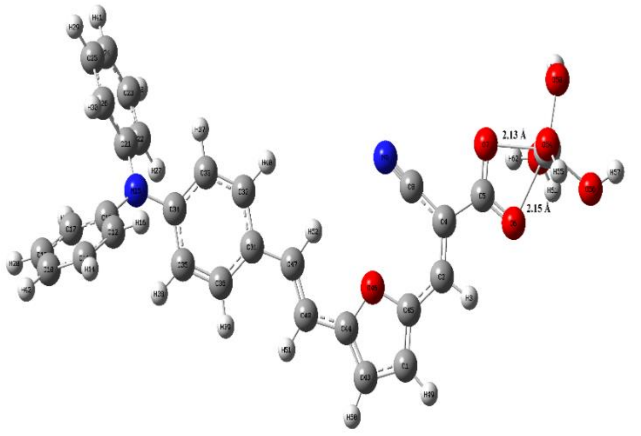

A0

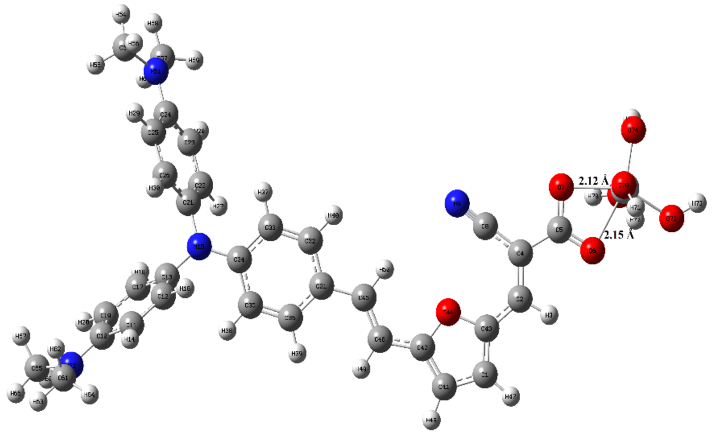

A1

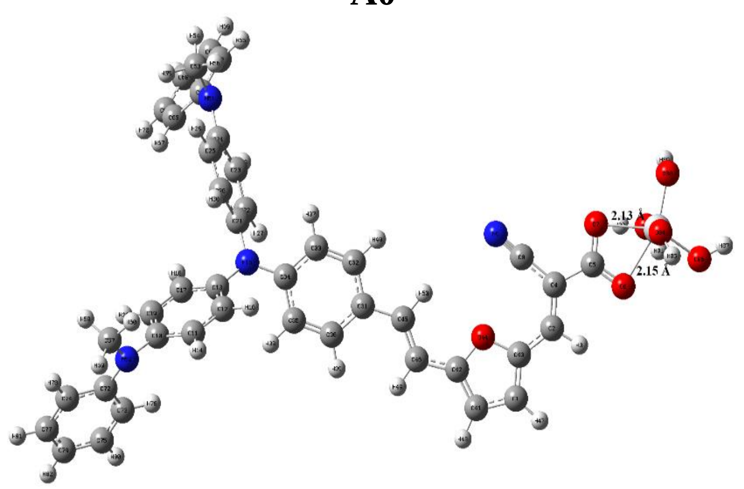

A2

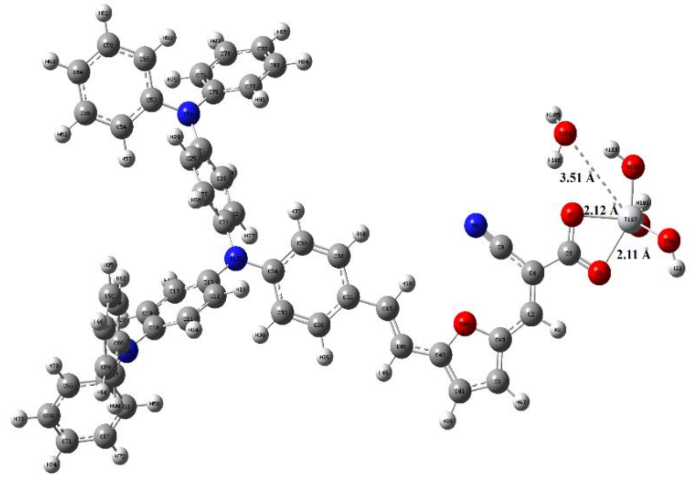

A3

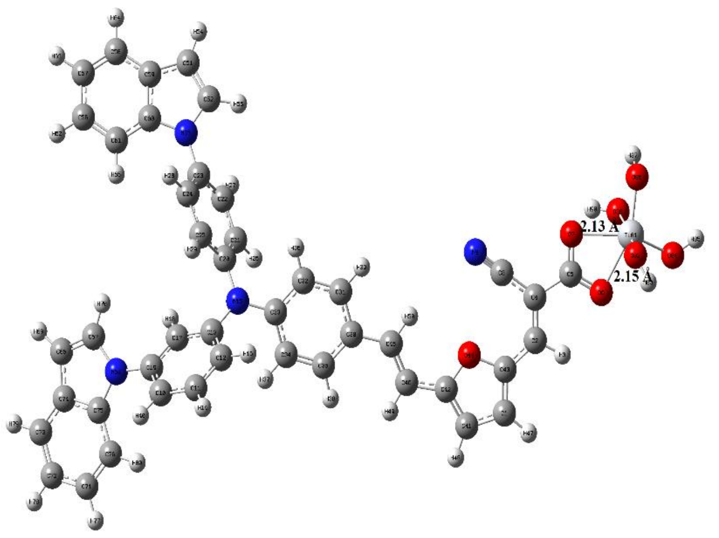

A4

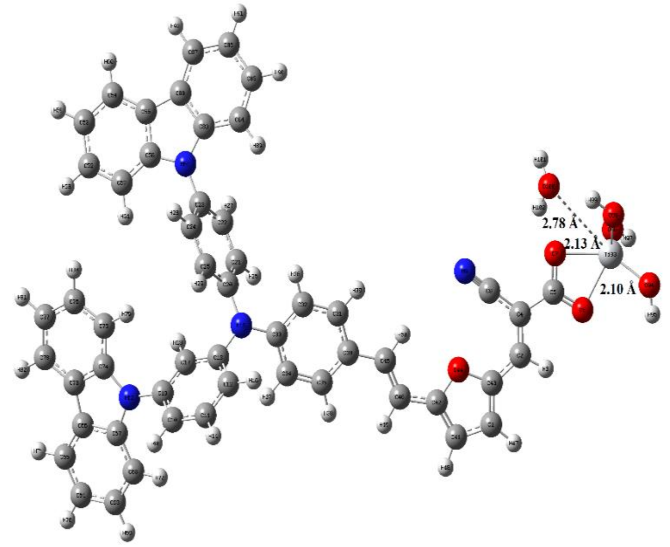

A5

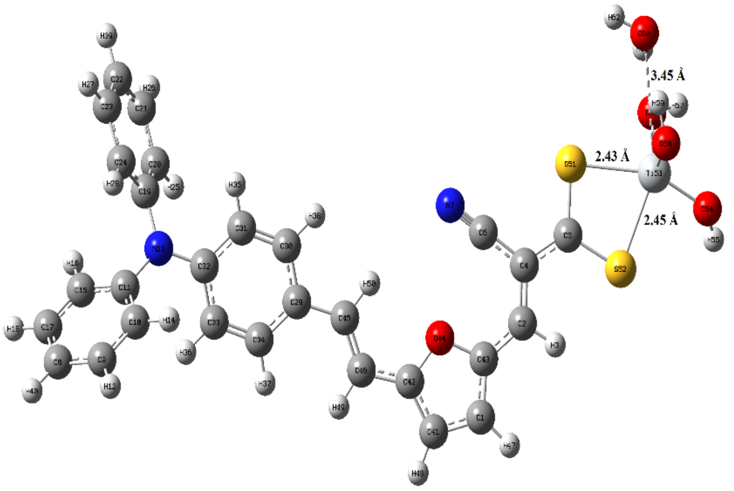

B0

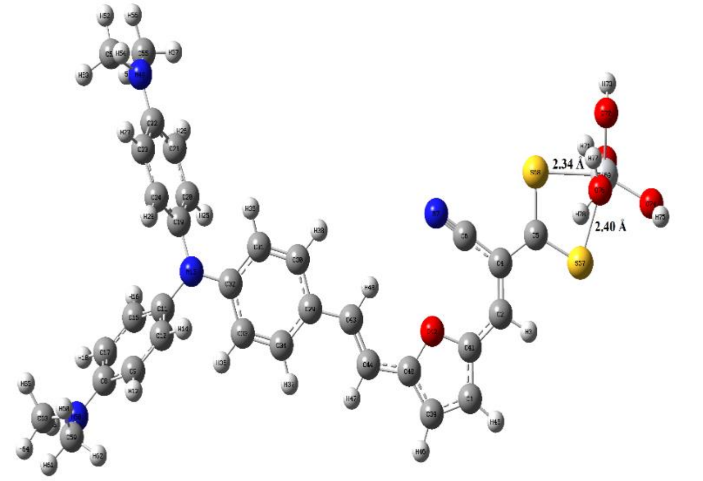

B1

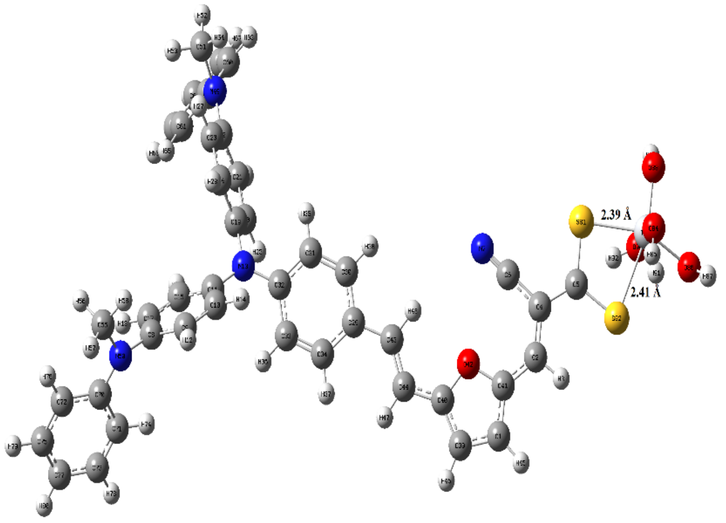

B2

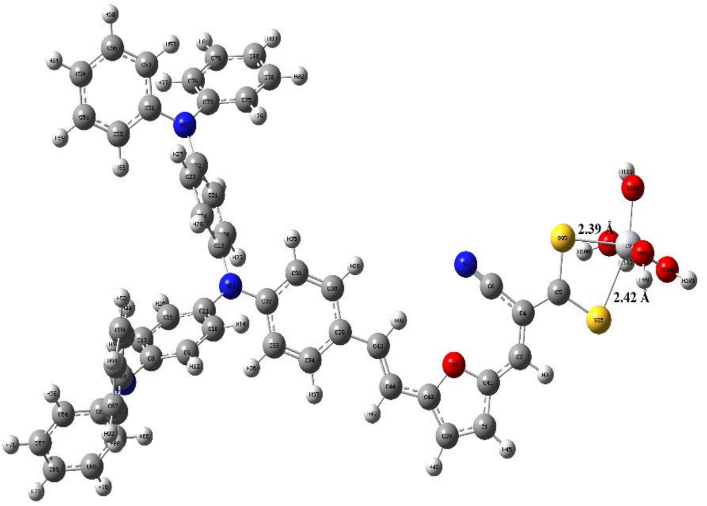

B3

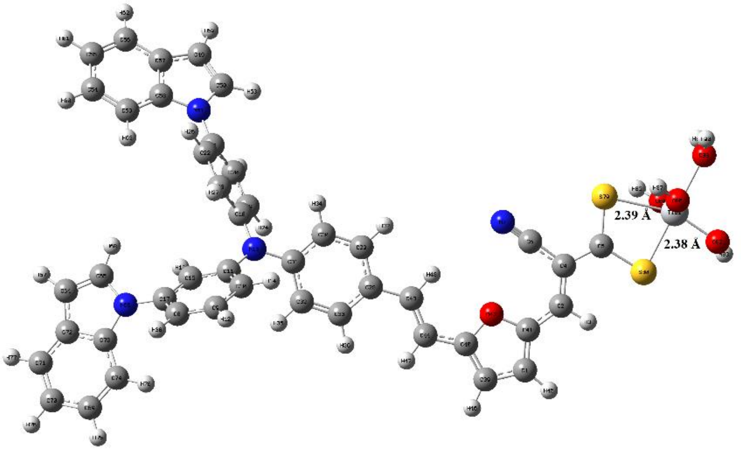

B4

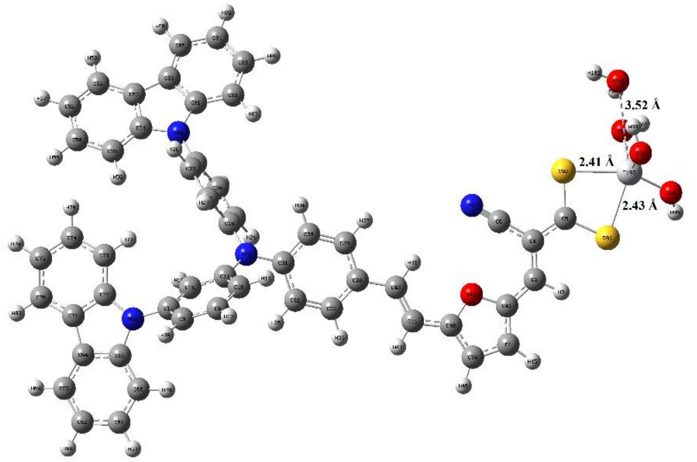

B5

Figure S1. The optimized geometry of all designed dyes attached with the $Ti(OH)_4$ surface.
